# miR-138-5p inhibits the malignant progression of prostate cancer by targeting FOXC1

**DOI:** 10.1186/s12935-020-01386-6

**Published:** 2020-07-09

**Authors:** Dapeng Zhang, Xiaodong Liu, Qingwei Zhang, Xin Chen

**Affiliations:** Department of Urology Surgery, Chifengshi Hospital, Chifeng, 024000 Neimenggu China

**Keywords:** miR-138-5p, FOXC1, Prostate cancer, Malignant progression

## Abstract

**Background:**

This study aimed to uncover the effect of miR-138-5p on the proliferation and metastasis of PCa cell lines, and further explore the potential regulatory mechanisms via regulating FOXC1.

**Methods:**

60 pairs cancer tissues and corresponding paracancerous ones from PCa patients were collected to assess the expression level of miR-138-5p by qRT-PCR. Subsequently, over-expression of miR-138-5p were established to explore the proliferation and metastasis of miR-138-5p in PCa cell lines was analyzed by CCK-8, Transwell assay and Wounding healing assay, respectively. Bioinformatics analysis and luciferase reporter gene assay were performed to search for the target genes of miR-138-5p, and FOXC1 was selected. Finally, the biological role of miR-138-5p and FOXC1 in the progression of PCa was clarified by a series of rescue experiments.

**Results:**

The results of qRT-PCR revealed that miR-138-5p was lowly expressed in PCa tissues and cell lines. Besides, the PCa patients with low-miR-138-5p had a high Gleason score, lymph node metastasis and poor prognosis of PCa, compared with these patients with high-miR-138-5p. Over-expression of miR-138-5p inhibited the proliferative, migratory and invasive capacities of PC-3 and DU-145 cells. Bioinformatics analysis and luciferase reporter gene assay suggested that FOXC1 was predicted to be the target gene of miR-138-5p. Moreover, FOXC1 expression level was negatively correlated to that of miR-138-5p in PCa tissues. Importantly, over-expression of FOXC1 could reverse miR-138-5p mimic induced-inhibition of PCa malignant progression.

**Conclusions:**

Downregulated miR-138-5p was closely associated with high Gleason score, more lymph node metastasis and poor prognosis of PCa patients. In addition, miR-138-5p alleviated the malignant progression of PCa by targeting and downregulating FOXC1.

## Background

Prostate cancer (PCa) is one of the most common malignancies all over the world, which is the leading cause of cancer-related deaths in the United States and European countries [1–3]. In recent years, the incidence and mortality rate of PCa in China has rapidly increased year by year, which has become a serious threat to human healthy [4, 5]. When prostate specific antigen (PSA) test was primarily used to screen for PCa before symptoms appear, the detection rate of PCa peaked in the early 1990s [6, 7]. So far, approximately 85% new diagnosed PCa cases were limited to early-stage cancer [8]. Although PSA test greatly improved the early-stage diagnostic rate of PCa, its benefit in decreasing the mortality of PCa remained controversial [9, 10]. Like other malignancies, the malignant progress of PCa is a multi-step and multi-stage process, including inactivation of anti-oncogenes and/or activation of proto-oncogenes [11, 12]. Currently, the target therapy based on cancer-related miRNAs presented a promising application, and the results of these studies showed that miRNA had a good application prospect in the diagnosis, treatment, prognosis and other aspects of cancer, to provide new ideas for the pathogenesis of PCa [13, 14].

MiRNAs are small, endogenous non-coding RNAs that negatively regulate expressions of protein-coding genes at translational level [15, 16]. MiRNAs exert the biological function by degrading or inhibiting translation of mRNAs [16]. It is reported that miRNAs are extensively involved in affecting cellular behaviors and progression of disease [17, 18]. Accumulating evidences have demonstrated the effects of abnormally expressed miRNAs on the malignant progression of tumors [17, 19]. These certain miRNAs might be utilized for developing anti-cancer drugs or biological hallmarks [20]. About 30% human genome could be regulated by miRNAs, and most of human miRNAs locate on cancer-associated genomic regions or gene fragile sites [21, 22]. MiR-138-5p is a newly discovered cancer-related miRNA, which has been confirmed to be down-regulated in pancreatic cancer, colorectal cancer and other malignant tumors [21, 22]. However, its expression and function of PCa are rarely reported.

Bioinformatics analysis has been widely applied for analyzing the target gene of miR-138-5p to further uncover the expression pattern of genes. By analyzing miR-138-5p profiling microarray, Forkhead box C1 (FOXC1) was selected to be the target gene of miR-138-5p. FOXC1 is an essential member of the forkhead box transcription factors and has been highlighted as an important transcriptional regulator of crucial proteins associated with a wide variety of carcinomas [23, 24]. Based on the above series of researches, this study aimed to elaborate the possible roles of miR-138-5p and FOXC1 in the progression of PCa, as well as the association with its clinical characteristics and prognosis of PCa patients, so as to bring a new idea for clinical diagnosis and treatment of PCa.

## Materials and methods

### Patients and PCa tissue samples

A total of 60 PCa patients undergoing radical prostatectomy were enrolled in this study from Department of Urology Surgery, Chifengshi hospital. PCa cancer tissues and corresponding paracancerous ones (5 cm away from tumor edge) were surgically resected and preserved within 5 min ex vivo. The clinical and pathological characteristics of PCa patients were collected for further analyses. The clinical stages of PCa were graded in accordance with International Federation of PCa staging criteria. In addition, the longest diameter of the tumor is measured and used to assess the tumor size. All patients in this study had been fully signed the informed consent. In addition, this study has been approved by the Ethics Committee of Chifengshi hospital.

### Cell lines and reagents

Prostate epithelial cell line (RWPE-1) and PCa cell lines (LNCaP, 22RV1, PC-3 and DU-145) were provided by ATCC, USA. Cells were cultured in RPMI 1640 or DMEM (Dulbecco’s modified MEM medium) containing 10% fetal bovine serum (FBS), penicillin (100 U/mL), and streptomycin (100 μg/mL) at 37 °C with 5% CO_2_.

### Transfection

Negative control (NC mimic) and miR-138-5p overexpression sequence vectors (miR-138-5p mimic) were purchased from GenePharma (GenePharma, Shanghai, China). After PCa cell lines were plated in 6-well plates and grown to a cell density of 40–60%, transfection was performed using Lipofectamine 3000 (Invitrogen, CA, USA) according to the manufacturer’s instructions. These cells were collected for verification of transfection efficacy and subsequent experiments after 48 h.

### CCK-8 assay

After 48 h of transfection, the cells were seeded in the 96-well plates with 2 × 10^3^ cells per well. After cultured for 24 h, 48 h, 72 h and 96 h, these cells were added with CCK-8 kit (Dojindo Laboratories, Japan). After incubation for 2 h, the fluorescent absorbance at the optical density (OD) value of 450 nm of each sample was recorded for plotting the proliferative curves in the microscope.

### Transwell assay

After 48 h of transfection, the cells were adjusted to a dose of 2.0 × 10^5^/mL. 200 μL suspension was applied in the upper side of Transwell chamber (Millipore, MA, USA) inserted in a 24-well plate. In the bottom side, 700 μL of medium containing 10% FBS was applied. After 48 h of incubation, these cells penetrated to the bottom side were fixed in 4% paraformaldehyde for 15 min, dyed with crystal violet for 20 min and counted using the microscope. The number of migratory cells was counted in five randomly selected fields per sample.

### Wound healing assay

After 48 h of transfection, these cells were inoculated in 6-well plates and grown to 90% confluence. After the creation of an artificial wound in cell monolayer, the medium with 1% FBS was replaced. After 24 h, the wound closure was captured by the microscope.

### Quantitative real-time PCR (qRT-PCR)

Total RNA was extracted from PCa cell lines and tissues using TRIzol reagent (Invitrogen, Carlsbad, CA, USA), purified by DNase I treatment, and reversely transcribed into cDNA by Primescript RT Reagent (Takara, Otsu, Japan). The obtained cDNA was subjected to qRT-PCR by SYBR^®^Premix Ex Taq™ (Takara, Japan). Data were normalized to GAPDH and U6. The following primers for qRT-PCR were used as followed:

miR-138-5p:

forward, 5′-GCGAGCTGGTGTTGTGAATC-3′,

reverse, 5′-AGTGCAGGGTCCGAGGTATT-3′;

U6:

forward, 5′-CTCGCTTCGGCAGCACA-3′,

reverse, 5′-AACGCTTCACGAATTTGCGT-3′;

FOXC1:

forward, 5′-CGGGTTGGAAAGGGATATTTA-3′,

reverse, 5′-CAAAATGTTCTGCTCCTCTCG-3′;

GAPDH:

forward, 5′-GAAATCCCATCACCATCTTCCAGG-3′,

reverse, 5′-GAGCCCCAGCCTTCTCCATG-3′.

### Western Blotting

The transfected PCa cell lines were lysed using PRO-PREPTM lysis buffer, shaken on ice for 30 min, and centrifuged at 14,000×*g* for 15 min at 4 °C. Total protein concentration was calculated by the BCA Protein Assay Kit (Pierce, Rockford, Il, USA). Rabbit anti-human monoclonal antibody against FOXC1 was purchased from Santa Cruz, USA; horseradish peroxidase-labeled goat anti-rabbit secondary antibody was purchased from Genscript. Data were normalized to GAPDH. Protein samples were separated by SDS-PAGE, transferred to PVDF membrane, and blocked with 5% skim milk powder for 1 h at room temperature. Primary antibody was added for incubation overnight at 4 °C shaker. In the next day, the membrane was rinsed 3 times with TBST and incubated with second antibody for 1 h at room temperature. After that, the protein samples on the membrane were finally semi-quantitatively analyzed by alpha SP image analysis software.

### Dual-luciferase reporter assay

3′-UTR of wild-type (WT) human FOXC1, which contains a putative miR-138-5p binding DNA sequence, was amplified by PCR and inserted into a p-miR-reporter (Ambion, USA) to create a firefly FOXC1-WT luciferase vector. The mutant (MUT) 3′-UTR was also inserted into p-miR-reporter to create a firefly FOXC1-MUT luciferase vector. Human HEK293T cells were transduced with NC mimic or miR-138-5p mimic, then cross-transfected with FOXC1-WT or FOXC1-MUT for 48 h. After that, the relative luciferase activities were measured using a Dual-luciferase reporter assay (Promega, USA) according to the manufacturer’s protocol.

### In vivo xenograft vectors

The Animal Ethics and Use Committee of Chifengshi hospital approved the cancer-forming experiment in nude mice. 8-week-old male nude mice were purchased from the animal center and randomly divided into two groups (5 in each group). The PC-3 cells with miR-138-5p mimic were injected subcutaneously into the axilla of mice. Tumor size was monitored every 7 days; Then, after 6 weeks, the mice were sacrificed. The tumor volumes were calculated using the following formula: tumor volume = (width^2^ × length)/2.

### Statistically analysis

GraphPad Prism 6 V6.01 was used for data analyses. Data were expressed as mean ± standard deviation, and *p* < 0.05 was considered as statistically significant. Intergroup differences were analyzed by the t-test. Kaplan–Meier curves were introduced for survival analysis. Chi-square test was performed to evaluate the correlation between miR-138-5p level and the pathological indexes of PCa patients.

## Results

### miR-138-5p was down-regulated in PCa tissues and cell lines

**Fig. 1:**
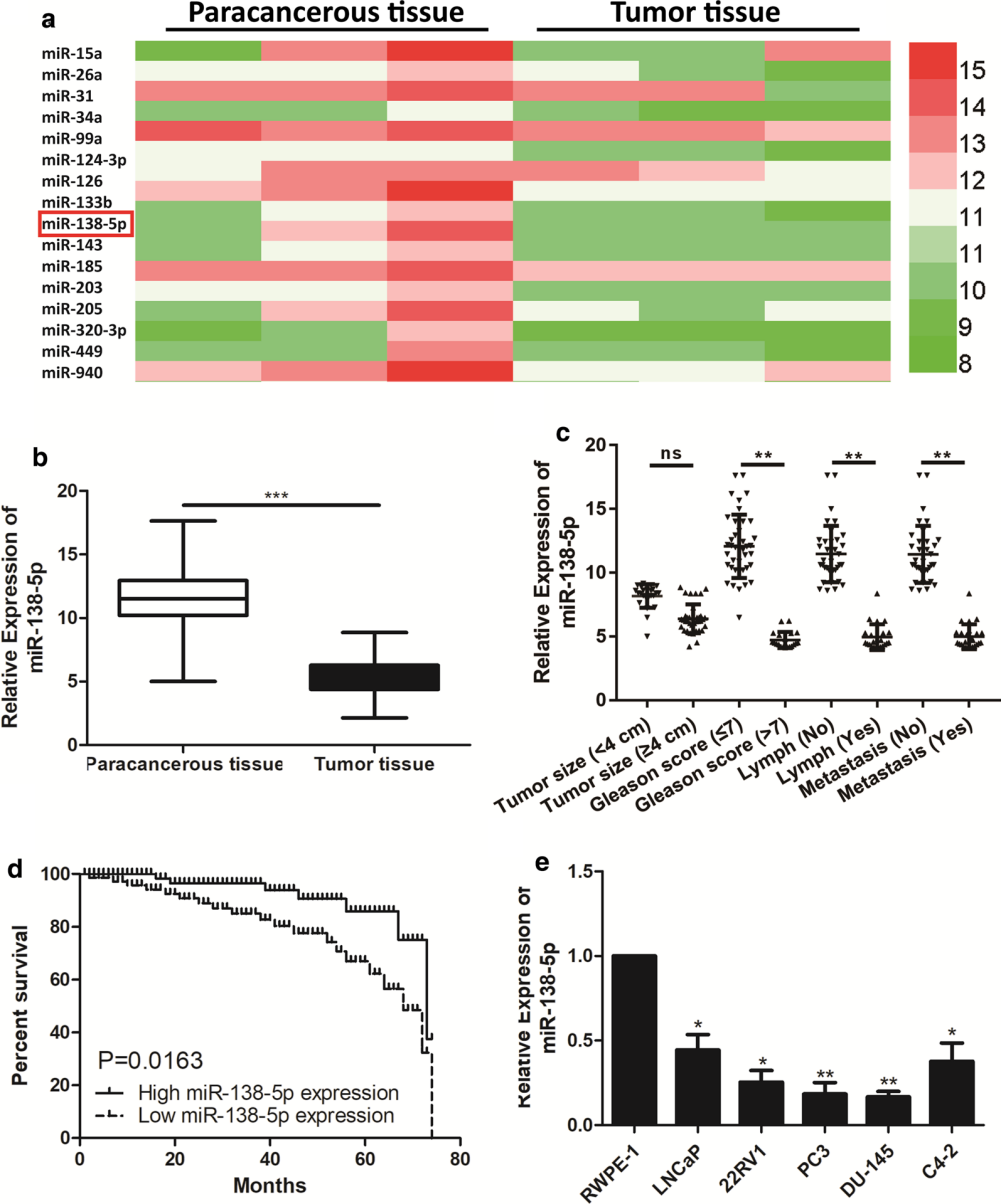
miR-138-5p is lowly expressed in PCa tissues and cell lines. **a** The heatmap of miRNAs expression profiles with PCa progression in TCGA database; **b** qRT-PCR was used to detect the expression level of miR-138-5p in PCa tissues and paracancerous tissues; **c** qRT-PCR was used to detect the difference expression of miR-138-5p in tissue samples of PCa patients with different clinicopathologic characteristics (tumor size, Gleason score, lymph node metastasis and bone metastasis); **d** Kaplan–Meier survival curve of PCa patients based on miR-138-5p expression; **e** qRT-PCR was used to detect the expression level of miR-138-5p in PCa cell lines. Data are mean ± SD, *p < 0.05, **p < 0.01, ***p < 0.001

Data from PCa patients of TCGA were complied for investigating the potential relevant miRNAs associated with the progression of PCa. We firstly focused insight into the expression level of miRNAs form TCGA database, and miR-138-5p was finally selected and was significant statistical difference in PCa tissues (Fig. 1a). qRT-PCR was performed to evaluate the expression of miR-138-5p in PCa tissues and cell lines. As showed in Fig. 1b, miR-138-5p was down-regulated in PCa tissues, compared with paracancerous tissues. Similarly, miR-138-5p was also down-regulated in PCa cell lines, compared with that of Prostate epithelial cell line (RWPE-1) (Fig. 1e).

### miR-138-5p expression was correlated with clinicopathologic characteristics and overall survival in PCa patients

The clinicopathology characteristics and follow-up data of enrolled PCa patients were collected for further analyses. According to the median level of miR-138-5p, PCa patients were assigned into two groups, including high-miR-138-5p level group and low-miR-138-5p level group. As shown in Table 1, the associations between the expression level of miR-138-5p and age, tumor size, Gleason score, lymph node metastasis and bone metastasis of PCa patients were analyzed. The results found that these PCa patients with low-miR-138-5p expression had a higher Gleason score and more lymph node metastasis in PCa patients, compared with these patients with high-miR-138-5p expression (Fig. 1c). In addition, Kaplan–Meier methods revealed the poor prognosis in PCa patients of low-miR-138-5p level group than that of high-miR-138-5p level group (Fig. 1d).

**Table 1:** Association of miR-138-5p expression with clinicopathologic characteristics of prostate cancer

Parameters	Number of cases	miR-138-5p expression		p-value
		High (%)	Low (%)	
Age (years)				0.830
< 60	24	14	10	
≥ 60	36	22	14	
Tumor size				0.526
< 4 cm	28	18	10	
≥ 4 cm	32	18	14	
Gleason score				*0.025*
≤ 7	40	28	12	
> 7	20	8	12	
Lymph node metastasis				*0.009*
No	37	27	10	
Yes	23	9	14	
Bone metastasis				0.109
No	35	24	11	
Yes	25	12	13	

### miR-138-5p alleviated the proliferation and metastasis of PCa cell lines

**Fig. 2:**
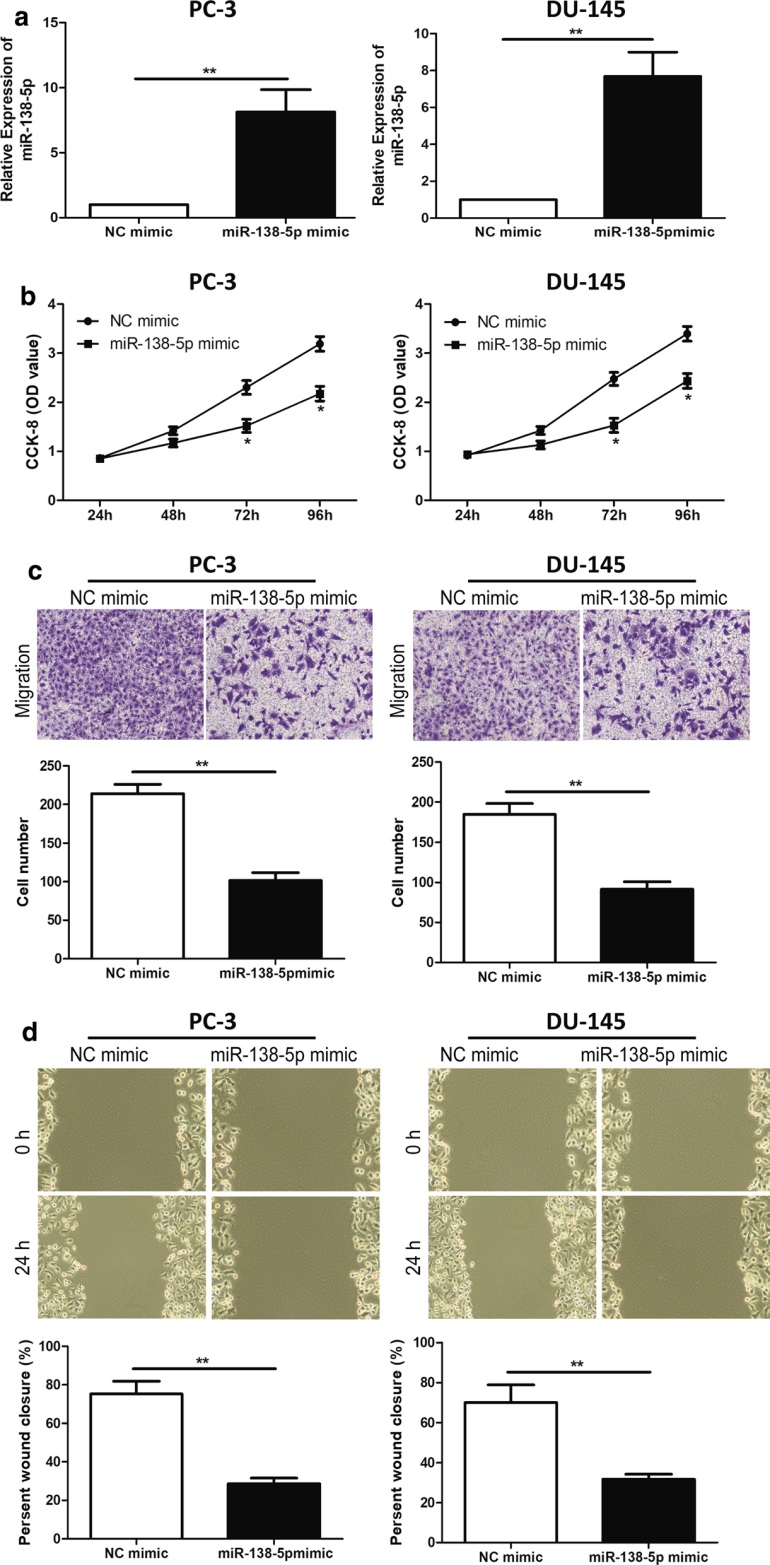
miR-138-5p inhibited the proliferation and metastasis of PCa cell lines. **a** qRT-PCR was used to verify the transfection efficiency of miR-138-5p after transfection of NC mimic and miR-450b-3p mimic in PC-3 and DU-145 cell lines; **a** CCK-8 assay detected cell proliferation of PCa cell lines after transfection of NC mimic and miR-138-5p mimic; **b** Transwell assay detected the migration of PCa cell lines after transfection of NC mimic and miR-138-5p mimic (magnification: 40×); **c** Wound healing assay detected the invasion and crawling ability of PCa cell lines after transfection of NC mimic and miR-138-5p mimic (magnification: 40×). Data are mean ± SD, *p < 0.05, **p < 0.01

To explore the biological function of miR-138-5p in PCa cell lines, the proliferation and metastasis of PCa was analyzed by CCK-8 assay, Transwell assay and Wounding healing assay, respectively. miR-138-5p mimic and NC mimic were successfully constructed in PC-3 and DU-145 cell lines, respectively (Fig. 2a). It was found by the CCK-8 assay that the cell proliferation ability of miR-138-5p mimic was significantly decreased in PCa cell lines, compared with that of NC mimic (Fig. 2b). Transwell assay revealed that the metastasis ability of PCa cells was significantly decreased in miR-138-5p mimic, compared with NC mimic (Fig. 2c). In addition, Wound healing assay also showed that the overexpression of miR-138-5p could hinder the invasion and crawling ability of PCa cell lines (Fig. 2d). These results suggested that miR-138-5p could inhibited the proliferation and metastasis in PCa cell lines.

### Interaction of miR-138-5p and FOXC1

**Fig. 3:**
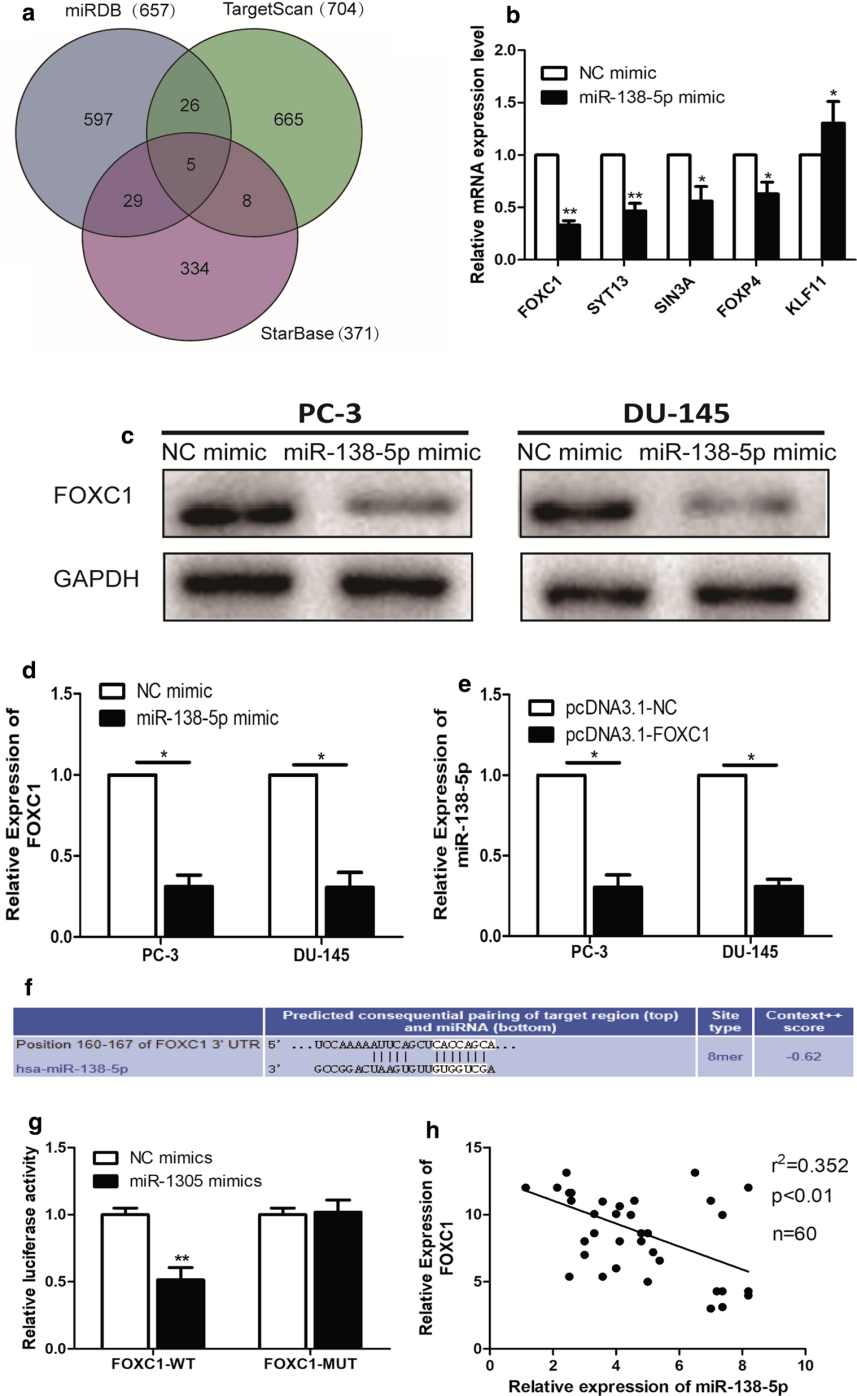
Interaction of miR-138-5p and FOXC1. **a** Bioinformatics analysis websites (miRDB, TargetScan and StarBase) showed the potential downstream target gene of miR-138-5p; **b** qRT-PCR was used to detect the differential expression of the potential downstream target gene of miR-138-5p in NC mimic and miR-138-5p mimic, respectively; **c** Western Blotting verified the expression level of FOXC1 after transfection of NC mimic and miR-138-5p mimic PCa cell lines, respectively; **d** qRT-PCR verified the expression level of FOXC1 after transfection of NC mimic and miR-138-5p mimic PCa cell lines, respectively; **e** qRT-PCR verified the expression level of miR-138-5p after transfection of pcDNA3.1-NC and pcDNA3.1-FOXC1 in PC-3 and DU-145 cell lines, respectively; **f**, **g** Dual luciferase reporter assays demonstrated direct targeting of miR-138-5p to FOXC1; **h** A significant negative correlation between miR-138-5p and FOXC1 expression in PCa tissues. Data are mean ± SD, *p < 0.05, **p < 0.01

Potential target genes of miR-138-5p were predicted in the miRDB, TargetScan and StarBase (Fig. 3a). At last, the intersection contained five potential targets (FOXC1, SYT13, SIN3A, FOXP4 and KLF11). Among them, FOXC1 was one of the most differentially expressed gene after the transfection of miR-138-5p mimic in PC-3 cells (Fig. 3b). Western Blotting showed that the expression level of FOXC1 was significantly down-regulated in PC-3 and DU-145 cells in miR-138-5p mimic, compared to NC mimic (Fig. 3c). In addition, the results of qRT-PCR also revealed the same trend (Fig. 3d). To further uncover the biological role of FOXC1 in PCa cell lines, we constructed FOXC1 overexpressing plasmid (pcDNA3.1-FOXC1) and empty overexpressing plasmid NC (pcDNA3.1-NC). qRT-PCR found that miR-138-5p level was found to be significantly down-regulated in PCa cell lines transfected with pcDNA-FOXC1, compared to that transfected with pcDNA-NC (Fig. 3e). Luciferase reporter assay verified that miR-138-5p could indeed combine with FOXC1 through specific sequences (Fig. 3f, g). Additionally, a significant negative correlation was identified to detect the expression levels of miR-138-5p and FOXC1 in PCa tissues (Fig. 3h).

### miR-138-5p negatively regulated FOXC1 to inhibit the malignant progression of PCa

**Fig. 4:**
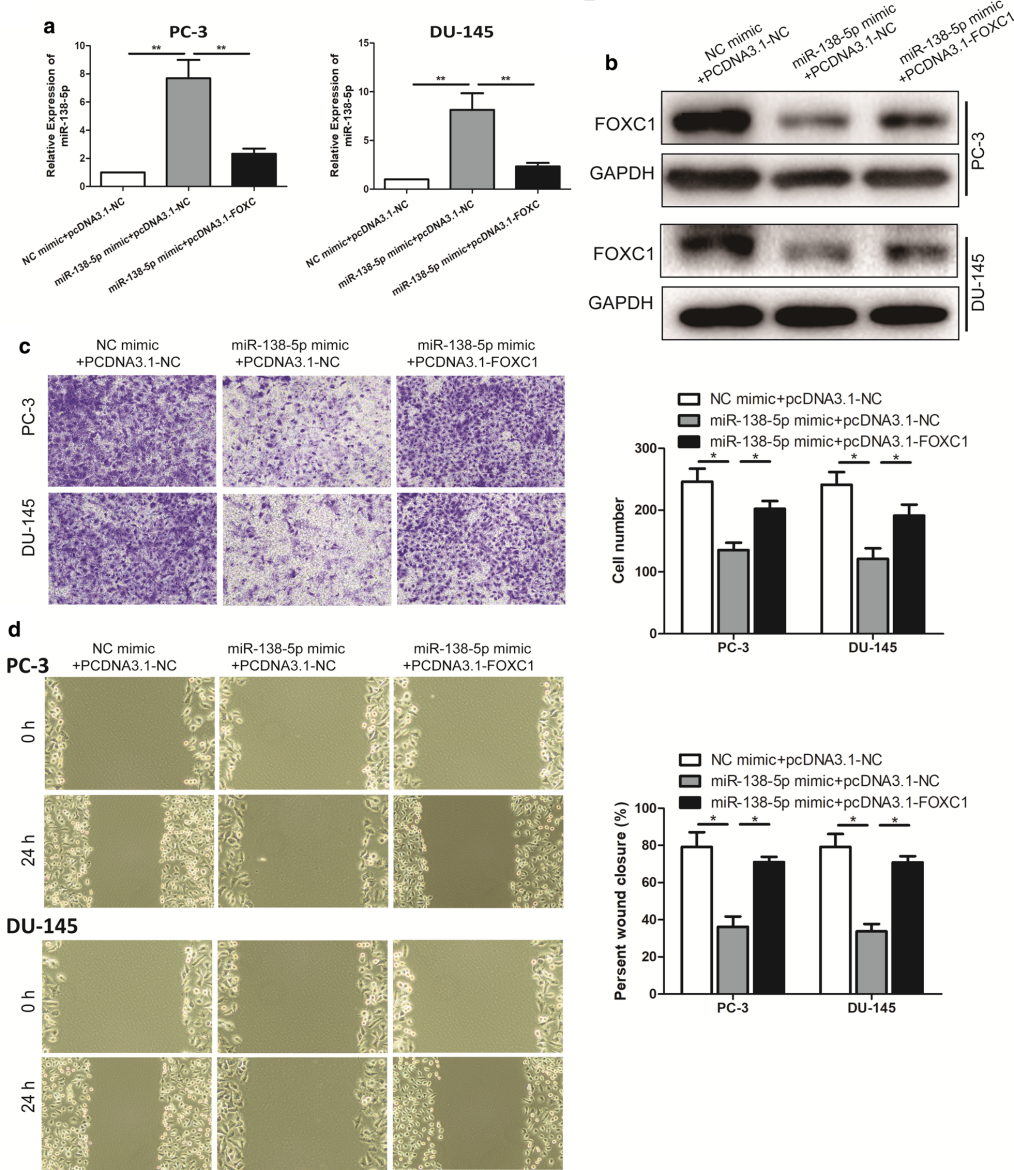
miR-138-5p negatively regulated the expression of FOXC1. **a** The expression level of miR-138-5p in the co-transfected PCa cell lines of miR-138-5p and FOXC1 was detected by qRT-PCR; **b** The expression level of FOXC1 in the co-transfected PCa cell lines of miR-138-5p and FOXC1 was detected by Western Blotting; **c** Transwell assay detected the cell migration in the co-transfected PCa cell lines of miR-138-5p and FOXC1 (magnification: 40×); **d** Wound healing assay was used to detect the invasion and crawling ability of PCa cell lines after co-transfection of miR-138-5p and FOXC1 (magnification: 40×). Data are mean ± SD, *p < 0.05, **p < 0.01

To further explore the regulatory mechanisms in which miR-138-5p exactly regulated FOXC1 to inhibit malignant progression of PCa. Firstly, the overexpressed endogenous FOXC1 was established with pcDNA3.1-FOXC1, and pcDNA3.1-NC to transfect PCa cell lines with miR-138-5p mimic. qRT-PCR demonstrated that PC-3 and DU-145 cells transfected with pcDNA3.1-FOXC1 had significantly lower miR-138-5p expression level than these cells transfected with pcDNA3.1-NC (Fig. 4a). In addition, Western Blotting also demonstrated that PC-3 and DU-145 cells transfected with pcDNA3.1-FOXC1 had significantly higher FOXC1 expression level than these cells transfected with pcDNA3.1-NC (Fig. 4b). Subsequently, overexpression of FOXC1 was demonstrated to be able to counteract the carcinostasis of miR-138-5p mimic on the occurrence of PCa by Transwell assay and Wound healing assay (Fig. 4c, d). Therefore, these results revealed that miR-138-5p could inhibit the malignant progression of PCa through modulating FOXC1.

### Over-expression of miR-138-5p suppressed the PCa in vivo tumorigenicity

**Fig. 5:**
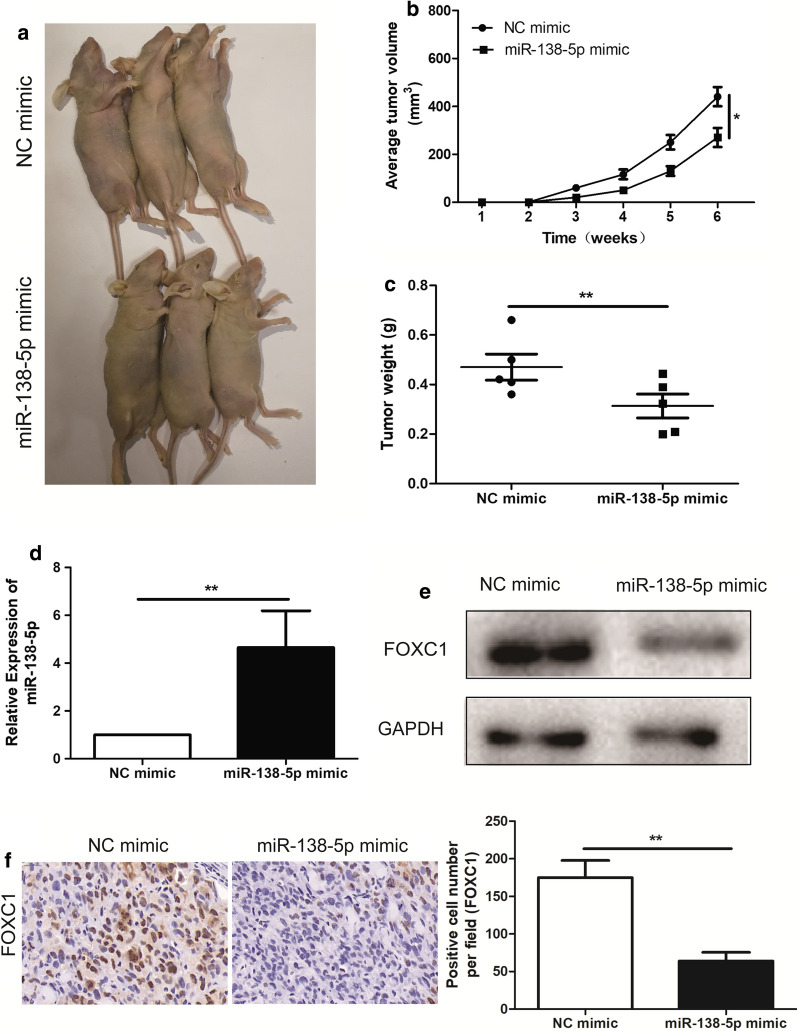
miR-138-5p inhibited tumorigenic ability in nude mice. **a**, **b** Tumor volume growth curves were calculated for different nude mice after injection of NC mimic and miR-138-5p mimic, respectively; **c** Tumor weight growth curves were calculated after injection of NC mimic and miR-138-5p mimic, respectively; **d** qRT-PCR was used to detect the expression level of miR-138-5p in the tumor-forming tissues of nude mice; **e** Western Blotting was used to detect the expression level of FZD4 in the tumor-forming tissues of nude mice; **f** Immunohistochemistry was used to detect the expression level of FZD4 in the tumor-forming tissues of nude mice with Hep3B cell line (magnification: 40×). Data are mean ± SD, *p < 0.05, **p < 0.01

In an in vivo tumorigenicity assay, NC mimic or miR-138-5p mimic transduced PC-3 cells were subcutaneously inoculated into the abdominal compartments of athymic nu/nu mice for 6 weeks. The volumes of PC-3 xenografts were calculated weekly. It showed that, in vivo tumor growth was significantly suppressed by miR-138-5p mimic, compared to NC mimic (p < 0.05; Fig. 5a, b). Subsequently, we validated the reduction of weight in the tissues of nude mice injected with miR-138-5p mimic (p < 0.05; Fig. 5c). The results of qRT-PCR revealed that miR-138-5p mimic in the tissues of nude mice could decrease the expression level of miR-138-5p (Fig. 5d). In addition, compared with NC mimic, FOXC1 expression significantly decreased in the tissues of nude mice with miR-138-5p mimic by Western Blotting (Fig. 5e). Immunohistochemistry showed that FOXC1 expression level of miR-138-5p mimic-transduced PC-3 xenografts significantly decreased than NC mimic-transduced xenografts (Fig. 5f).

## Discussion

The etiology of PCa is complex, and no clear results about the pathogenesis of PCa is found [1, 7, 9]. Nowadays, the understanding of the pathogenesis and biological behavior of PCa still has great limitations [8, 9]. The development of PCa is a multi-factors process, influenced by a variety of biomolecules and regulated by signaling pathways [11, 12]. With the progress of the Human Genome Project, most researches on molecular and cellular pathobiology have been extensively conducted to detect the differential expression profile of tumor genes, which is of great significance for exploring the molecular mechanism of PCa, and finding the molecular biomarkers in the early diagnosis and prognosis of PCa [13, 14].

MiRNAs not only participate in normal physical biological processes, but also regulate cancer progression at transcriptional and post-transcriptional levels [15, 16]. It is estimated that over 30% of genes in cellular processes are regulated or controlled by miRNAs [17, 20]. In the past years, tumor-promotor or tumor-suppressor miRNAs were used to be as drug targets for the treatment of PCa [15, 18, 20]. Nowadays, cancer-related miRNAs have been well concerned, exerting more crucial application in early-stage diagnosis, treatment and prognosis of cancer [13, 14, 19]. As a member of cancer-associated miRNAs family, miR-138-5p is located on chromosome Xq38.13 [25]. Previous researches showed that miR-138-5p could inhibit the malignant progression of some human cancer, such as pancreatic cancer and colorectal cancer [25, 26]. In this study, miR-138-5p was down-regulated in PCa tissues and cell lines. Besides, these PCa patients with low-miR-138-5p expression had a high Gleason score, more lymph node metastasis, and poor prognosis of PCa patients, compared with these patients with high-miR-138-5p expression. Thus, the above results suggested that miR-138-5p might act as anti-cancer effect in the proliferation and metastasis of PCa. In order to further investigate the biological function of miR-138-5p in PCa cell lines, CCK-8, Transwell assay and Wounding healing assay were used to introduce that miR-138-5p mimic could inhibit the proliferation and metastasis of PCa. The above results provided a theoretical basis for revealing the mechanism of the development of PCa. Of course, the specific molecular mechanism of signal transduction in PCa need to further study in the subsequent studies.

Regulatory mechanism of miRNAs depends on the expression and function of the related target genes [15, 20]. A miRNA degrades target mRNA or suppress its translation by base pairing with 3′UTR of the mRNA [15, 16]. The degree of base pairing decides the degradative or translation inhibitory effect of a miRNA, that is, complete base pairing leads to mRNA degradation; otherwise, translation inhibition is achieved [27, 28]. MiRNAs only account for only 1% of the whole human genome, but are able to regulate more than 30% protein-encoding genes [29]. Multiple miRNAs could precisely regulate a single target gene [16, 22]. Bioinformatics analysis and luciferase reporter gene assay showed that FOXC1 was the target gene of miR-138-5p through predicting in the miRDB, TargetScan and StarBase. FOXC1 expression level was up-regulated in PCa tissue and cell lines. Based on these findings, we hypothesized that miR-138-5p might act as a ceRNA in the malignant progression of PCa and found that miR-138-5p negatively regulated FOXC1 expression in PCa tissues. In addition, qRT-PCR showed that compared with NC mimic, miR-138-5p mimic could decreased the expression level of FOXC1. In order to explore the associations between miR-138-5p and FOXC1 in the development of PCa, the overexpression of FOXC1 was found to reverse the proliferation and metastasis ability of miR-138-5p mimic on PCa cell lines, thus promoting the malignant progression of PCa.

## Conclusions

In summary, the down-regulated miR-138-5p was closely associated with high Gleason score, more distant metastasis and poor prognosis of PCa patients. In addition, miR-138-5p alleviated the malignant progression of PCa by targeting and downregulating FOXC1.

## Data Availability

The datasets used in this study are available from the corresponding author upon reasonable request.
